# Use of Adipose-Derived Mesenchymal Stem Cells in Keratoconjunctivitis Sicca in a Canine Model

**DOI:** 10.1155/2015/527926

**Published:** 2015-02-23

**Authors:** Antonio J. Villatoro, Viviana Fernández, Silvia Claros, Gustavo A. Rico-Llanos, José Becerra, José A. Andrades

**Affiliations:** ^1^Laboratory of Bioengineering and Tissue Regeneration (LABRET), Department of Cell Biology, Genetics and Physiology, Faculty of Sciences, Biomedical Research Institute of Málaga (IBIMA), University of Málaga, 29071 Málaga, Spain; ^2^Networking Biomedical Research Center in Bioengineering, Biomaterials and Nanomedicine (CIBER-BBN), Spain; ^3^Centro Andaluz de Nanomedicina y Biotecnología (BIONAND), University of Málaga, 29590 Málaga, Spain

## Abstract

Keratoconjunctivitis sicca (KCS) or dry eye disease (DED) is an immune-mediated multifactorial disease, with high level of prevalence in humans and dogs. Our aim in this study was to investigate the therapeutic effects of allogeneic adipose-derived mesenchymal stromal cells (Ad-MSCs) implanted around the lacrimal glands in 12 dogs (24 eyes) with KCS, which is refractory to current available treatments. Schirmer tear test (STT) and ocular surface integrity were assessed at 0 (before treatment), 3, 6, and 9 months after treatment. Average STT values and all clinical signs showed a statistically significant change (*P* < 0.001) during the follow-up with reduction in all ocular parameters scored: ocular discharge, conjunctival hyperaemia, and corneal changes, and there were no signs of regression or worsening. Implanted cells were well tolerated and were effective reducing clinical signs of KCS with a sustained effect during the study period. None of the animals showed systemic or local complications during the study. To our knowledge, this is the first time in literature that implantation of allogeneic Ad-MSCs around lacrimal glands has been found as an effective therapeutic alternative to treat dogs with KCS. These results could reinforce a good effective solution to be extrapolated to future studies in human.

## 1. Introduction

Keratoconjunctivitis sicca (KCS) or dry eye disease (DED) is one of the most commonly encountered problems in ophthalmology in humans and dogs. The current prevalence of dry eye in the world is estimated around 5% to 35% [1–3] and between 4 and 20% in canine specie [4] with important health and economic implications. KCS is a multifactorial disease with dysfunction in a component of the lacrimal functional unit, leading to changes in the volume, composition, or clearance of the tear film, which results in symptoms of discomfort, visual disturbances, and tear film instability with potential damage to the ocular surface. Although their mechanisms are not yet completely understood, there is enough evidence suggesting a cytokine and receptor-mediated inflammatory process affecting both the lacrimal gland and the ocular surface, with progressive immune-mediated destruction of lacrimal tissue in humans and dogs [5, 6].

The current treatment protocols in KCS are difficult, last lifetime with variability in efficacy and safety [7, 8].

Adipose-derived mesenchymal stromal cells (Ad-MSCs) are multipotent stem cells with capacity to differentiate into osteogenic, adipogenic, chondrogenic, myogenic, and many other cell lineages with important secretory faculties of different bioactive molecules with trophic, paracrine, and immunomodulatory functions [9–13]. Mesenchymal stem cells (MSCs) express low levels of major histocompatibility complex class I (MHC-I) but lack expression of MHC-II surface molecules and thereby cannot serve as effective antigen-presenting cells to promote immune responses on various activated lymphoid cells, including T cells, B cells, natural killer cells, and dendritic cells [12, 14]. Their low immunogenicity and their immunoregulatory potential allow their allogeneic use, which makes them an alternative to be a promising new treatment for severe refractory autoimmune diseases [9, 15]. They have been extensively studied as a cellular therapy for different pathologic conditions, with the dog as an animal model [16].

The dog is considered to be a superior animal model of KCS, because dogs develop the disease naturally [4] and they have great similarities with humans [17]. The most common cause of dry eye is an immune-mediated inflammatory response targeted against tears producing glands.

The aim of our study was to evaluate the safety and the therapeutic effects of local implantation of allogeneic Ad-MSCs around the lacrimal glands in dogs considering different ocular clinical parameters during a 9-month follow-up where current standard treatments of KCS were ineffective.

## 2. Materials and Methods

All animal procedures and protocols were conducted by licensed veterinary surgeons and comply with both national and European legislation (Spanish Royal Decree RD1201/2005 and EU Directive 86/609/CEE as modified by 2003/65/CE, resp.) for the protection of animals used for research experimentation and other scientific purposes.

### 2.1. Animals

Twelve dogs client-owned of different breeds, 4 females and 8 males, aged between 4 and 12 years and weighing from 9 to 40 kg were selected. All individuals were affected by bilateral KCS at least for 6 months. They were refractory to conventional treatments (corticosteroids, tacrolimus, cyclosporine, and artificial tears), with a Schirmer tear test (STT) <10 mm/min in each eye, and without any viable therapeutic alternatives. Dogs did not receive any kind of anti-inflammatory or immunomodulatory medications for at least two weeks before treatment; they were kept with artificial tears only.

Untreated or placebo animals were not included in the present study, since only dogs with naturally occurring KCS and ineffective treatment response were selected.

All dog owners signed a written consent before initiation of this experimental procedure and were fully informed that long-term outcome, safety, complications, and efficacy of the cell implantation in KCS were not known.

### 2.2. Clinical Evaluation

All animals underwent a periodical full veterinarian clinical and ophthalmic examination. To assess the clinical course we measured the tears production and evaluated ocular surface health of each eye. Tears production was measured using the STT at one minute [18]. The ocular surface health was assessed with the clinical measurements of ocular discharge (OD), conjunctival hyperaemia (Hyp), and corneal changes (CC) (edema, vascularization, pigmentation, and corneal ulcer), graded as absent (0), mild (1), moderate (2), or severe (3). All these parameters were collected and measured before treatment (baseline) and 3, 6, and 9 months after cell implantation.

### 2.3. Isolation and Culture of Ad-MSCs

Adipose tissue was aseptically collected from gluteal subcutaneous fat in the hip under general anesthesia with isoflurane (Isovet, B Braun Vetcare) of three healthy male dogs, 2 years old, and weighing 18 +/− 1,5 kg, through a small surgical incision and maintained at 4°C in a tube with culture medium.

Under a laminar flow hood, after weighing the harvested adipose tissue, 5 grams of fat was minced and mixed with 20 mL of Hanks solution (Sigma-Aldrich, Madrid, Spain) containing 0,1% collagenase type II (Sigma-Aldrich) by incubating at 37°C for 90 minutes in orbital agitation. After digestion, the cell suspension was filtered through a 100 *μ*m cell strainer. The cell suspension was centrifuged at 400 g for 5 minutes to discard the lipid layer and the obtained cell pellet was washed with culture medium. Primary cultures were carried out in T175 flasks with Dulbecco's modified Eagle's medium (DMEM) containing 10% (v/v) fetal bovine serum (FBS), 2.5 mM L-glutamine, 100 U/mL penicillin, 100 *μ*g/mL streptomycin, and 1.25 *μ*g/mL fungizone (all from Sigma-Aldrich). The culture medium was changed 2 times per week and the cells were selected by their capacity to attach to the flask surface, discarding the floating cells in the first medium change at 72 hours. When culture flasks became 80% confluence, cells were detached with 0.25% trypsin containing 1 mmol/L EDTA and subsequently replated at a concentration of 10^4^ cells/cm^2^ for continued passaging. The remaining cells were cryopreserved in cryopreservation media (10% dimethyl sulfoxide and 90% FBS), frozen at −80°C in an isopropanol jacketed closed container (Nalgene Cryo freezing container), and stored in liquid nitrogen until the next day. All experiments and* in vivo* implantation were conducted at passage 2.

### 2.4. Cell Yield, Population-Doubling Time, and Cell Proliferation

Cell yield was determined from the number of cells obtained in primary culture at semiconfluence (p0) relative to one fat gram of the processed sample.

Population-doubling time (PDT) was determined in continual subculture and growth of Ad-MSCs harvested at semiconfluence at passages 1 and 2 and was calculated using the formula *x* = ln⁡⁡(*N*/*No*)/ln⁡⁡(2), where *N* is the final cell number and *No* is the initial cell number at the beginning of the logarithmic increase.

Cell proliferation was measured using MTS (CellTiter 96 Aqueous One Solution Cell Proliferation Assay, Promega) assay according to manufacturer protocol. Briefly, in a 96-well plate, 3000 Ad-MSCs per well were seeded using 8 wells as replicates of each sample. The cells were allowed to proliferate performing the change of medium twice a week and making readings on days 1, 4, 7, 11, 14, 18, and 21. Supernatants were collected and absorbance was measured at 490 nm using a microplate reader (ELx800, Bio-Tek instruments).

### 2.5. Flow Cytometry Analysis

Fluorescence-activated cell sorting (FACS) was used to characterize canine Ad-MSCs from passage 2. Cultured cells were trypsinized, spun down in a centrifuge, and washed twice in FACS buffer consisting of 10 mM hepes (Gibco/Invitrogen), 100 U/mL penicillin, 100 *μ*g/mL streptomycin, and 2 mg/mL bovine serum albumin (BSA) (Sigma-Aldrich) in Leibovitz's L-15 medium (Gibco/Invitrogen). After the washing step, cells aliquots (1 × 10^6^ cells) were incubated in FACS buffer containing fluorochrome-conjugated monoclonal antibodies against CD29, CD90 and STRO-1 (R&D Systems, Minneapolis, MN, USA), CD34 and CD45 (MiltenyiBiotech SL), and MHC-II (BD Pharmingen, Becton Dickinson), or an appropriate isotype control antibody (Sigma-Aldrich). After 30 minutes in the dark on ice, cells were washed again in FACS buffer before analysis. Five hundred thousand events per sample were analyzed on a*MoFlo* SP1338 (DakoCytomation, Denmark) using Summit software. Cells were gated on forward and side scatter to exclude debris and cell aggregates, and dead cells were excluded by 7-aminoactinomycin D (7-AAD, BD Pharmingen) staining.

### 2.6. *In Vitro* Multilineage Cell Differentiation

To assess the multipotentiality, Ad-MSCs at passage 2 were differentiated along adipogenic, osteogenic, and chondrogenic lineages according to standard protocols, as follows.

For adipogenic differentiation, six-well culture plates were seeded at a density of 2 × 10^4^ cells/cm^2^ and cultured in DMEM 10% FBS to confluence. Then, medium was completely replaced with adipogenic induction medium consisting of DMEM 10% FBS supplemented with 15% (v/v) rabbit serum (Gibco), 10^−7^ M dexamethasone, 0.5 mM 3-isobutyl-1-methylxanthine (IBMX), 10 mg/mL bovine insulin, and 0.2 mM indomethacin (all from Sigma-Aldrich). Equal cell number was cultured in DMEM 10% FBS for negative control. Media were changed every 3-4 days. Adipogenic differentiation was evaluated by Oil red O staining on day 21 after induction. Briefly, the culture medium was discarded, and cells were washed three times with PBS and fixed with 4% paraformaldehyde for 15 minutes at room temperature. Then each well was washed with distilled water and incubated with Oil Red O solution (1 mg/mL in 60% isopropanol). After 15 minutes at room temperature, cells were rinsed with distilled water and lipid vacuoles appeared red.

For osteogenic differentiation, cells were seeded in six-well plates at a density of 3 × 10^3^ cells/cm^2^ and cultured in DMEM 10% FBS until reaching 80% confluence. The complete medium was then replaced with osteogenic differentiation medium consisting of DMEM 10% FBS supplemented with 10^−8^ M dexamethasone, 50 *μ*M ascorbate-2-phosphate, and 10 mM *β*-glycerophosphate (all from Sigma-Aldrich). As a negative control an equal number of wells were cultured in DMEM 10% FBS. Media were changed twice a week. Alkaline phosphatase (ALP) activity and Alizarin Red S staining for detecting calcium deposition were assessed on day 21 after induction.

ALP was assessed using staining kit (Sigma-Aldrich). Briefly, cells were rinsed twice with 1 mL of Tyrode's balanced salt solution (TBSS, Sigma-Aldrich) and then fixed in citrate buffered acetone for 30 seconds at room temperature. The fixed cells were rinsed with distilled water and then incubated in dark conditions for 45 minutes with 1 mL of the alkaline-dye mixture (12 mg Fast Violet B salt and 2 mL Naphthol AS-MX phosphate alkaline solution in 48 mL of distilled water). After incubation, the cells were washed with distilled water and observed under inverted phase-contrast microscopy. In this assay, a red color at the plasma membrane indicates the expression of ALP.

Alizarin Red S staining was performed for detecting calcium deposition. In brief, cells were rinsed twice with 1 mL of TBSS and fixed in 70% ethanol for 1 hour at 4°C. After three washes with distilled water, cells were stained with 1 mL of 2% Alizarin Red S (pH 4.2, Sigma-Aldrich) for 10 minutes in dark conditions. All stained cells were observed under inverted phase-contrast microscopy after washing twice with TBSS.

For chondrogenic differentiation, a 3D pellet culture model was used. Briefly, pellets were formed in 15 mL polypropylene conical tubes by centrifugation to 400 g 5 min of 2.5 × 10^5^ cells suspended in 0.5 mL of serum-free medium consisting of DMEM high glucose (4.5 g/L) supplemented with 10^−7^ M dexamethasone, 0.12 mM ascorbate-2-phosphate (all from Sigma-Aldrich), 1% ITS+ Premix Tissue Culture Supplement, 1 mM sodium pyruvate (both from Gibco/Invitrogen), and 10 ng/mL recombinant human transforming growth factor-beta 1 (rhTGF-*β*1, R&D Systems). Pellet culture without growth factor supplementation served as the negative control for this study. Full media changes were performed every other day. On day 21 pellets were fixed in 10% of neutral buffered formalin, dehydrated in alcohol, embedded in paraffin, and sectioned at 10 *μ*m thick. Subsequently, the sections were stained with toluidine blue (TB) that exhibits metachromatic reaction with cartilage matrix.

### 2.7. Cell Transplantation

The procedure was performed under ultrashort intravenous anesthesia with propofol 6 mg/kg (Propofol Lipuro 1%, B Braun Vetcare).

Twenty-four eyes of 12 selected animals with KCS were implanted aseptically with one injection of 5 × 10^6^ Ad-MSCs in 0.4 mL DMEM, using a 20 G needle around main lacrimal gland, and one injection of 3 × 10^6^ cells surrounding the gland of the third eyelid, with preliminary vitality test with trypan blue staining. Dogs continue with artificial tears as it was previously to implantation.

### 2.8. Statistics

All data are represented as the mean values ± standard deviations for every ocular parameter collected from the evaluated 24 eyes, which are considered as independent samples. Since the data was not normally distributed, statistical analyses were performed using the Kruskal-Wallis test and the all pairwise multiple comparisons between groups were performed with a Tukey test. For cell proliferation data, comparisons were done using Student's *t*-test (*P* < 0.05). A *P* value less than 0.05 was considered significant. All analyses were carried out using SigmaPlot 13.0.

## 3. Results

### 3.1. Isolation and Culture of Ad-MSCs

**Figure 1:**
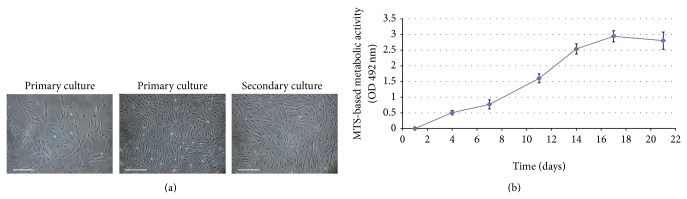
Cell morphology and proliferative curve of canine Ad-MSCs cultures. (a) In primary cultures, a large number of adherent cells with fibroblastic morphology were observed from the first day of culture, forming frequent CFUs. On secondary culture, canine Ad-MSCs appeared as spindle-shaped cells that were grown in a monolayer. Bars 200 *μ*m. (b) Representative curve obtained with MTS cell proliferation assay at p2 showing from 24 hours an important proliferative capacity, initiating the logarithmic growth phase, and reaching its plateau phase around 14 days.

The subcutaneal fat pads from each dog were processed and the isolation of MSCs was successful in all donor samples. Canine Ad-MSCs were isolated using their ability to adhere to tissue culture plastic. In primary cultures, a large number of colony-forming units (CFUs) consisted of cells with fibroblastic morphology were observed 2 days after initial seeding and 80% of confluence was reached at day 6 (Figure 1(a)). On secondary cultures, canine Ad-MSCs appeared as spindle-shaped cells that were grown in a monolayer and maintained their proliferation capacity without indication of senescence.

### 3.2. Cell Yield, Population-Doubling Time, and Cell Proliferation 

The number of Ad-MSCs obtained per gram of adipose tissue sample of each animal donor at passage p0 was of 15.6 × 10^6^, 17.1 × 10^6^, and 14.9 × 10^6^ cells/g, mean value 15.87 × 10^6^ ± 1.12 cells/g.

The population-doubling time at passage p1 was 2.35, 2.4, and 2.54 days, mean value 2.43 ± 0.098 days, and 2.54, 2.75, and 2.9 days, mean value 2.757 ± 0.07 days at passage p2 for each donor. All samples were considered similar because there were no statistically significant differences between these groups (*P* < 0.05).

The curve obtained with MTS cell proliferation assay showed at 24 hours an important proliferative capacity, initiating the logarithmic growth phase and reaching its plateau phase around 14 days (Figure 1(b)).

### 3.3. Flow Cytometry Analysis

**Figure 2:**
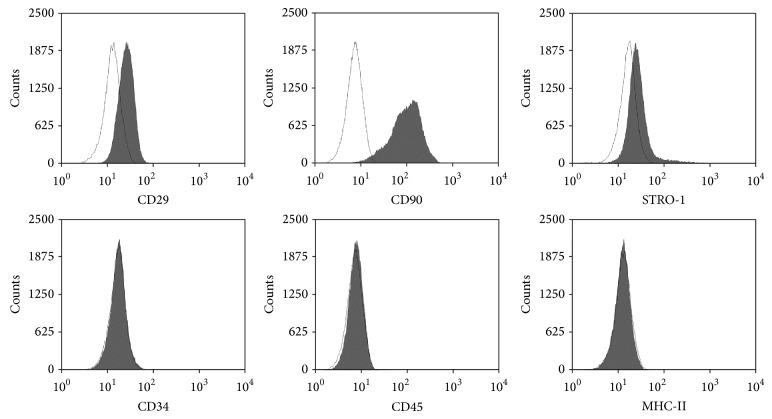
Representative immunophenotype profiles of canine Ad-MSCs for several mesenchymal and hematopoietic markers. FACS analysis revealed a homogeneous cell population, characterized by the positive expression of CD29, CD90 and STRO-1, and lack expression of CD34, CD45 and major histocompatibility class II (MHC-II).

Once the secondary cultures at passage 2 reached 70% of confluence, cells were subjected to FACS analysis for mesenchymal and hematopoietic markers. The profiles of canine Ad-MSCs revealed a homogeneous cell population, where positive cells for mesenchymal markers (CD29, CD90, and STRO-1) were found (Figure 2). Additionally, these cells were negative for the expression of hematopoietic markers CD34, CD45, and MHC-II.

### 3.4. *In Vitro* Multilineage Cell Differentiation

**Figure 3:**
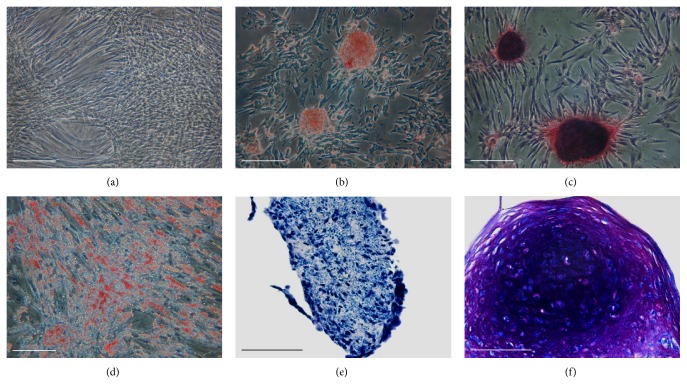
Assessment of trilineage differentiation. (a) Control. Cells maintained in control medium for 21 days. (b-c) Assessment of osteogenic differentiation. (b) Histochemical localization of ALP. Osteoinduced cells formed numerous nodules highly positive for ALP staining. (c) Positive Alizarin Red S staining by day 21 whereas red calcium nodules clearly appeared on the osteoinduced cultures. (d) Assessment of adipogenic differentiation. Positive Oil Red O staining by day 21 confirmed the presence of lipid droplets only in adipogenic-induced cells. (e-f) Assessment of chondrogenic differentiation. Histological sections of pellets after 21 days in the presence or absence of rhTGF-*β*1. (e) Control pellets incubated without rhTGF-*β*1. (f) Pellets incubated with rhTGF-*β*1 clearly displayed improved chondrogenesis with positive toluidine blue staining. Bars 200 *μ*m in (a–d) and 100 *μ*m in (e-f).

Adipogenic differentiation was confirmed by Oil Red O staining. After culturing cells with adipogenic-inducing media for 21 days, red-stained lipid droplets were present in the cytoplasm (Figure 3(d)).

Under osteogenic conditions for 21 days, cells formed white nodule-like aggregations, which were strongly stained for ALP activity and Alizarin Red S (Figures 3(b) and 3(c), resp.). Latter indicated that they were mineralized during the differentiation period. Nonsupplemented cells showed the spindle-shaped morphology and remained negative for ALP and Alizarin Red S staining (Figure 3(a)).

For chondrogenic differentiation, a 3D pellet culture model was used. Histological study showed that canine Ad-MSCs were able to differentiate into chondrocytes. After 21 days of culture, pellets incubated with rhTGF-*β*1 exhibited metachromasia when stained with TB, indicating a cartilaginous matrix (Figure 3(f)).

### 3.5. Cell Transplantation

Twenty-four eyes of 12 dogs with KCS were implanted with allogeneic canine Ad-MSCs. There were eight males and four females. The average age of dogs treated was 8,1 ± 2.5 years. There were no local or systemic complications during all follow-up periods. Individual parameters of each eye are detailed in Table 1, showing STT and clinical signs scored at pretreatment (baseline) and at 3, 6, and 9 months after implantation.

**Figure 4:**
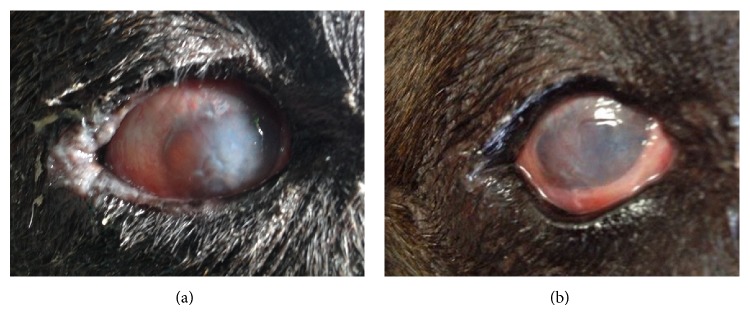
Photographs of the right eye of dog number 7 with chronic KCS and STT 0 mm/min during 2 years, severe ocular discharge, severe hyperaemia, and moderate cornea changes (edema, cornea opacity, and vascularization). (a) Eye at baseline (0). (b) Same eye at 9 months after cell implantation. STT 19 mm/min, without secretion, mild hyperaemia, and improvement on cornea changes and showing a good lacrimal meniscus.

The clinical observation of the process showed a significant improvement during the first three months after transplantation. This recovery remains stable until the last follow-up and did not show signs of regression or worsening (Figure 4).

**Figure 5:**
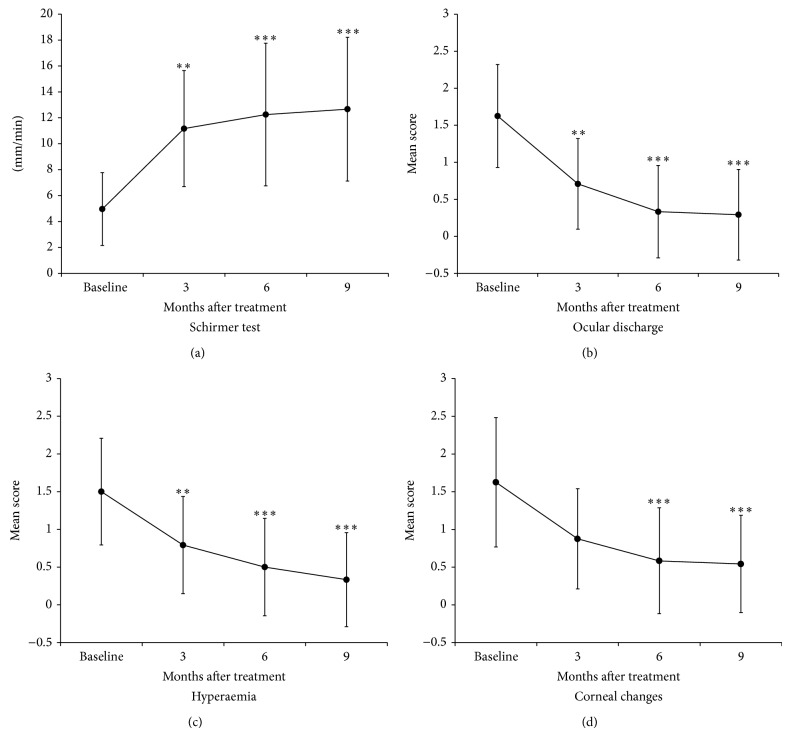
Mean values ± standard deviations of the ocular parameters scored during the 9-month follow-up after treatment. For Schirmer test, tears production was measured at different time points (a). For (b), (c), and (d) mean scores are represented. Stars indicate statistically significant differences in comparison with baseline. *P* < 0.01 (∗∗), and *P* < 0.001 (∗∗∗). There were no differences among the other groups in any case.

For STT, the baseline mean value was 4.96 ± 2.97 (Figure 5(a)). This value increased significantly (*P* < 0.01) to 11.16 ± 4.57 mm/min at 3rd month and it kept rising to 12.25 ± 5.62 mm/min at 6th month and to 12.66 ± 5.69 at 9th month. These last two values are significantly different from the baseline (*P* < 0.001). In spite of this rise between 3rd and 9th month, there were no statistical differences among these samples.

The scores obtained from the follow-up of the ocular discharge, hyperaemia and corneal changes, showed a sustained decrease over all the time period (Figures 5(b)–5(d)). These reductions were statistically significant between baseline and 6th and 9th month after treatment for all parameters (*P* < 0.001). Differences at 3rd month were significant for ocular discharge (*P* < 0.01), hyperaemia (*P* < 0.05) but there were no differences for corneal changes at 3rd month.

## 4. Discussion

This is the first clinical case report in a major animal model with KCS refractory to current conventional treatment that evaluates clinical results after implantation of allogeneic Ad-MSCs around lacrimal glands with a 9-month follow-up. KCS in dogs is an autoimmune disease that results in destruction of the lacrimal gland similar to those described in humans [5, 6].

The importance of this study is that the DED of the dog is a natural animal model of KCS. The dogs develop this disease naturally, and their clinical profiles are considerably more severe than in humans [4]. For this reason dogs with KCS are very valuable compared to other animal models [19], in terms of developing cell-based regenerative strategies.

The immunopathogenesis of DED is not fully understood; however, it is recognized that inflammation has a prominent role in the development of the symptoms and signs of dry eye. The hallmarks of lacrimal gland inflammation are the presence of immune cell infiltrates, loss of acinar epithelial cells (secreting cells), and increasing production of proinflammatory cytokines with inadequate tears secretion [20]. Secondly, factors that affect the homeostatic balance of the ocular surface system induces tears hyperosmolarity, activating intracellular signaling pathways that initiate the production of proinflammatory cytokines [5], elevated levels in the tears of patients with DED [21]. Those cytokines will activate migration of lymphocyte cells into the ocular surface with more secretion of inflammatory cytokines, chemokines, metalloproteinases, and proangiogenic molecules that will facilitate the infiltration of pathogenic immune cells that give rise to the clinical picture [5]. Studies in animal models have shown that proinflammatory cytokines also inhibit neurotransmitter release leading to insufficient lacrimal gland secretion [20].

The current treatment for dry eye, typically consisting of anti-inflammatory agent and/or immunosuppressant, has to be used for lifetime and is ineffective in some patients [6, 22, 23]. Besides, these agents are associated with deleterious and undesirable side effects that limit the long-term use of these drugs [5, 24].

In our study, we used adipose tissue because it is an easily affordable and plentiful source for obtaining MSCs with a high proliferation capacity. Canine Ad-MSCs are multipotent stem cells with capacity to differentiate, with important secretory faculty of different bioactive molecules with trophic, paracrine, anti-inflammatory, and immunomodulatory functions [10–12]. Ad-MSCs have been isolated and characterized from human and several animal species including dogs [25–27] and are currently being used as therapeutics for a number of clinical applications including ophthalmological disorders [28, 29].

As KCS is primarily a disease that mainly affects older patients with concomitant diseases [2], we decided to use allogeneic cells. Aging and different diseases are known to have a negative impact on the regenerative capacity of Ad-MSCs including changes in differentiation potential, proliferation ability, gene expression, angiogenic capacity, and immunomodulation [30–33]. The effective use of allogeneic MSCs in patients is possible due to their low immunogenicity, allowing a rapid initiation of therapy without the need for harvesting MSCs from each patient, screening the best donors, preventing transmission of infectious diseases, and evaluating* in vitro* their MSC profile and immunosuppressive features [12, 34].

Canine Ad-MSCs obtained from our donors have shown consistency in their isolation, expansion, high ratio proliferation, plastic adherent, and behavior* in vitro*, exhibiting its ability of adipogenic, osteogenic, and chondrogenic differentiation similar to those described for this specie [16, 25].

Our FACS data are consistent with other studies [16, 27, 35], although immunophenotyping the canine cells involves difficulty because of the limited availability of antibodies which cross-react with the dog [12]. The expression profile corresponding with those is widely described in the literature for MSCs [11, 12, 36], as shown by our results: positive expression of CD29, CD90 and STRO-1, and lack of expression of hematopoietic markers CD34, CD45, or MHC-II.

As far as we know, STRO-1 expression has been tested in MSCs from canine bone marrow tissue [37], but never in canine Ad-MSCs cultures, and this is the first study that describes it. STRO-1-positive population has been shown to be capable of differentiating into multiple mesenchymal lineages, including hematopoiesis-supportive stromal cells with a vascular smooth muscle-like phenotype, adipocytes, osteoblasts, and chondrocytes [38]. Lack of MHC-II has allowed us to confirm the low immunogenicity [12, 16].

All animals were transplanted in culture passage 2, allowing us to obtain the necessary amount of Ad-MSCs and avoid unnecessary additional subculturing, related with multipotential and proliferation rate decays, senescence, cell size increases, and chromosomal instabilities [39, 40].

Even with the great variability of the group of patients (gender, age, and weight) this study shows a substantial clinical improvement as well as statistically significant changes in mean scores for all parameters measured. This recovery remained stable until the last follow-up and showed no signs of regression or worsening. None of the animals showed systemic or local complications during the study, as it has been documented for long-term studies in domestic species which shown no adverse effects with the administration of MSCs in a large number of animals [12].

Our results are quite encouraging considering that we have treated animals with serious KCS refractory to current conventional treatments where there was no viable alternative to solve their pathology. If individual animals are revised, nine of twelve cases (9/12) have achieved a STT and ocular parameters very similar to normal values (normal STT mean values are 20.2 +/− 3.0) [18] during the follow-up.

We believe that the possible mechanism of the Ad-MSCs in KCS is based on their immunomodulatory and anti-inflammatory capacity, stimulated by proinflammatory cytokines released in the process (TNF, IFN, and IL-6) [5, 21] and through secretion of immunomodulatory soluble factors as TGF-*β*, HGF, PGE2, and IDO [11]. This effect is enough to break the vicious cycle of production of proinflammatory cytokines and lacrimal lymphocyte proliferation, reactivating tears production to initiate the repair of the ocular surface, perhaps promoting regeneration of the acinar epithelial cells of the lacrimal gland. This immunomodulatory capacity has been shown in other glandular immune-mediated disorders [41].

Furthermore, the prolonged clinical effect in our study could be explained by the persistency of the main cell population around the periorbital tissues and lacrimal gland area for more than 4 weeks like has been demonstrated [34]. So that is why we suggest to perform a second implantation between 1 and 3 months in cases in which there are no enough clinical improvements in the first 3 months (dogs 2, 3, and 12), since the therapeutic effects may not be sufficient after the first implantation.* In vivo* results indicate that MSCs could be administrated multiple times without eliciting a cellular immune response [12, 34].

From our results, we demonstrated that Ad-MSCs lacrimal gland implantation is a simple, safe, and effective treatment for KCS refractory to current treatments, with a clinical significant improvement, that allow removing much of the medication that must be applied for lifetime to these patients with KCS, improving the economic cost and quality of life. However, the present study has its limitations. Control group was not included, and the number of eyes treated was small. We recognize the need of future studies to include advanced technologies to measure tears composition [42].

In conclusion, our results indicate that allogeneic Ad-MSCs implantation around the lacrimal glands is a safe, effective, and relatively simple therapy of KCS in dogs, with a significant improvement of tears production and in all ocular clinical signs associated with the disease.

## Figures and Tables

**Table 1:** Demographics, STT values, and scores of ocular health.

Dog	Sex	Age	Time (months)	STT	Ocular discharge	Hyperaemia	Corneal changes
1	M	12	0	8/7	2/2	1/1	1/2
			3	14/12	1/1	1/1	1/1
			6	14/11	0/0	0/1	0/1
			9	15/12	0/0	0/0	0/0

2	M	8	0	0/4	2/2	2/2	2/2
			3	2/4	1/1	2/1	1/1
			6	1/4	1/1	2/1	1/1
			9	2/4	1/1	2/1	1/1

3	F	11	0	8/6	1/1	1/1	0/2
			3	13/9	0/1	0/1	0/1
			6	15/10	0/0	0/1	0/0
			9	15/10	0/0	0/0	0/1

4	M	4	0	9/6	1/2	2/2	1/2
			3	18/12	0/0	0/1	0/1
			6	16/14	0/0	0/1	0/1
			9	16/13	0/0	0/1	0/1

5	M	6	0	3/0	2/3	2/3	1/1
			3	14/12	1/1	1/1	0/1
			6	16/16	0/0	0/0	0/0
			9	18/16	0/0	0/0	0/0

6	F	6	0	7/6	2/1	2/2	0/1
			3	14/14	1/1	1/1	0/1
			6	18/17	0/1	0/1	0/0
			9	18/17	0/1	0/0	0/0

7	M	9	0	0/7	3/1	3/2	2/1
			3	12/16	1/0	2/0	2/0
			6	16/19	0/0	1/0	2/0
			9	19/20	0/0	1/0	2/0

8	M	6	0	5/4	1/1	1/1	3/3
			3	14/10	0/1	0/0	1/2
			6	16/11	0/1	0/0	1/2
			9	16/11	0/1	0/0	1/2

9	M	10	0	0/3	3/1	2/1	2/1
			3	3/7	2/0	2/1	2/0
			6	2/6	2/0	2/1	2/0
			9	3/7	2/0	2/0	2/0

10	M	8	0	7/9	1/1	1/1	3/3
			3	14/16	0/0	1/0	2/1
			6	17/17	0/0	0/0	1/0
			9	17/17	0/0	0/0	1/0

11	F	6	0	6/7	1/1	1/1	2/1
			3	14/14	1/0	1/0	1/0
			6	14/16	0/0	0/0	0/0
			9	14/16	0/0	0/0	0/0

12	F	11	0	3/4	2/2	1/0	1/2
			3	4/5	1/2	1/0	1/1
			6	3/4	1/2	1/0	1/1
			9	4/4	1/2	1/0	1/1

M: male; F: female; STT: Schirmer tear test in mm/min. Ocular discharge, hyperaemia, and corneal changes were graded as absent (0), mild (1), moderate (2), or severe (3). The right and left eye data were collected at baseline (0) and 3, 6, and 9 months after cell implantation.
